# Chronic stress-induced LC-NE dysfunction: cellular and axonal mechanisms underlying impaired neuromodulation

**DOI:** 10.3389/fnins.2026.1940938

**Published:** 2026-09-16

**Authors:** Mats Ericson

**Affiliations:** 1 Division of Ergonomics, Department of Biomedical Engineering and Health Systems, School of Engineering Sciences in Chemistry, Biotechnology and Health, KTH Royal Institute of Technology, Stockholm, Sweden; 2 Division of Surgery and Oncology, Department of Clinical Science, Intervention and Technology (CLINTEC), Karolinska Institutet, Stockholm, Sweden

**Keywords:** axonal transport, chronic stress, locus coeruleus, mitochondrial dysfunction, neuromodulation, norepinephrine

## Abstract

The locus coeruleus-noradrenergic (LC-NE) system is a central neuromodulatory network involved in regulation of arousal, attention, and adaptive behavioral function. Chronic stress has been associated with altered LC activity, including shifts in tonic-phasic regulation, yet the direction, magnitude, and persistence of these alterations appear to depend on stress paradigm, duration, and context, and the biological mechanisms underlying the emergence and persistence of this dysregulated state remain incompletely understood. Here, we examine how prolonged stress exposure may contribute to LC-NE dysfunction through interacting cellular and axonal mechanisms. Prolonged corticotropin-releasing factor (CRF)-mediated activation is proposed to impose a chronic tonic load on LC neurons, increasing metabolic demand, calcium burden, and mitochondrial oxidative stress. Because LC neurons combine autonomous pacemaking activity with exceptionally extensive axonal arborization, prolonged stress exposure is likely to promote cumulative cellular strain, impaired intracellular transport, and reduced efficiency of distal norepinephrine signaling. Within this model, chronic stress is conceptualized as producing a loss of dynamic regulatory capacity—a reduced ability of the LC-NE system to shift flexibly between tonic and phasic modes of signaling—rather than simply a quantitative increase or decrease in norepinephrine levels, even in the absence of overt neuronal loss. Disruption of tonic-phasic signaling balance, reduced phasic responsiveness, impaired autoregulatory function, and diminished neuromodulatory flexibility could collectively contribute to persistent alterations in network regulation and cognitive function. This perspective reframes stress-related dysfunction as a progressive, cumulative process rather than a transient alteration in stress signaling alone. By linking chronic stress exposure to LC-NE dysfunction through integrated cellular and axonal mechanisms, this work provides a mechanistic basis for understanding persistent stress-related alterations in brain function and highlights restoration of adaptive LC-NE regulation as a potential therapeutic principle. The proposed framework integrates systems-level stress physiology with cellular and axonal neurobiology into a unified model of stress-induced LC-NE dysfunction.

## 1 Introduction

The locus coeruleus-noradrenergic (LC-NE) system constitutes a central neuromodulatory network with widespread projections throughout the brain, enabling coordinated regulation of neural activity across neural circuits (Aston-Jones and Cohen, 2005; Grimm et al., 2024; Poe et al., 2020; Ross and Van Bockstaele, 2021).

Through its influence on arousal, attention, and adaptive behavioral responses, the LC-NE system contributes to broad regulation of brain state and behavioral adaptation (Sara, 2009; Schwarz et al., 2015). While these functional properties have been extensively described, recent reviews have also examined stress-induced LC-NE neuroplasticity, the involvement of the LC-NE system in stress-related psychopathology and resilience, and LC vulnerability in neurodegenerative disease (Borodovitsyna et al., 2018; Farahani et al., 2025; Slavova et al., 2024). However, the interactions between cellular and axonal mechanisms in the development and persistence of chronic stress-related LC-NE dysfunction remain incompletely understood.

Previous reviews have approached these questions from complementary perspectives, including stress-induced neuroplasticity within the LC-NE system (Borodovitsyna et al., 2018), LC-NE function in stress-related psychopathology and resilience (Slavova et al., 2024), and LC vulnerability in neurodegenerative disease (Farahani et al., 2025). Building on this literature, the present review focuses specifically on how sustained stress-related LC activation may interact with cellular metabolic and mitochondrial strain and the demands imposed by extensive axonal arborization. The aim is to examine how these interacting processes may contribute to persistent impairment of dynamic LC-NE regulation.

Throughout this review, “chronic stress” refers to prolonged or repeated stress exposure without sufficient recovery, resulting in sustained engagement of central stress pathways. The term primarily encompasses mental or affective stress associated with persistent engagement of CRF-related stress systems (Borodovitsyna et al., 2018) as distinct from transient acute stress responses that are generally adaptive and followed by recovery. This usage is consistent with the concept of allostatic load, whereby repeated or sustained activation of stress systems imposes cumulative physiological burden over time (McEwen, 2007).

From this perspective, the term ‘rigidity' is used here as a conceptual descriptor of reduced neuromodulatory flexibility, referring to a state in which the capacity of the LC-NE system to adapt its activity to changing behavioral and environmental demands becomes constrained (Aston-Jones and Cohen, 2005; Grimm et al., 2024). Depending on the stress paradigm, duration, stage, and experimental context, this reduced flexibility may be expressed through different combinations of elevated tonic activity, reduced phasic responsiveness, and narrowing of the system's dynamic range rather than as a single uniform physiological state (Curtis et al., 2012; Slavova et al., 2024; Valentino and Van Bockstaele, 2008). This conceptualization is consistent with systems-level models emphasizing tonic-phasic balance and adaptive LC-NE regulation (Aston-Jones and Cohen, 2005; Grimm et al., 2024).

The locus coeruleus is a small nucleus located in the dorsal pontine brainstem, comprising a relatively limited number of neurons but giving rise to one of the most extensive projection systems in the central nervous system (Aston-Jones and Cohen, 2005; Poe et al., 2020). The LC-NE system is characterized by highly extensive noradrenergic projections extending throughout large portions of the brain, including cortex, hippocampus, amygdala, thalamus, cerebellum, and spinal cord (Chandler et al., 2014; Schwarz et al., 2015; Swanson and Hartman, 1975). This anatomical organization enables the LC-NE system to exert a global modulatory influence across neural systems rather than mediating spatially precise, point-to-point signaling (Bouret and Sara, 2005; Sara, 2009; Schwarz et al., 2015).

A key feature of this system is that noradrenergic transmission is not confined to classical synaptic communication. Instead, a substantial proportion of norepinephrine release occurs via volume transmission, in which neurotransmitter is released from axonal varicosities and diffuses within the extracellular space to act on both synaptic and extrasynaptic receptors (Agnati et al., 2010). Through this mechanism, the LC-NE system operates as a neuromodulatory system capable of influencing network excitability and state-dependent processing over large spatial domains (Agnati et al., 2010; Aston-Jones and Cohen, 2005; Sara, 2009). While localized synaptic signaling contributes to more specific effects in certain regions, diffuse transmission enables the system to influence brain function at a systems level. Functionally, LC neurons dynamically regulate neuromodulatory tone through interacting tonic and phasic modes of activity, which together support adaptive behavioral and cognitive flexibility (Aston-Jones and Cohen, 2005; Grimm et al., 2024; Rajkowski et al., 1994).

Recent projection-specific evidence indicates that LC projections may show distinct norepinephrine response profiles during arousal transitions. Matosevich et al. (2025) demonstrated partial anatomical segregation between LC neurons projecting to the pontine reticular nucleus (PRN) and those projecting to the basal forebrain, together with distinct norepinephrine response patterns during sensory-evoked arousal transitions. In particular, a rapid increase in norepinephrine signaling from the LC to the PRN was associated with sensory-triggered awakening and rapid transitions from sleep to wakefulness. These findings provide evidence for projection-specific differences in norepinephrine signaling during arousal transitions, while not directly demonstrating independently regulated phasic LC firing across projection systems (Matosevich et al., 2025).

Theoretical accounts have emphasized the importance of tonic-phasic balance for effective neuromodulation and adaptive behavioral regulation (Aston-Jones and Cohen, 2005; Bouret and Sara, 2005; Grimm et al., 2024). Within this model, chronic stress may bias LC activity toward less adaptive modes of neuromodulatory regulation, although the direction and magnitude of tonic-phasic alterations may vary with stress paradigm, duration, stage, and experimental context. Multiple cellular, molecular, and circuit-level mechanisms contributing to persistent stress-related LC dysregulation have been identified; however, their relative contributions and interactions in determining vulnerability, persistence, and resilience remain incompletely understood (Borodovitsyna et al., 2018; Slavova et al., 2024).

Existing models have characterized LC-NE dysfunction at functional, cellular, and circuit levels (Borodovitsyna et al., 2018; Slavova et al., 2024), while cellular and structural constraints associated with sustained physiological load have also been examined in LC neurons and other highly arborized neuromodulatory systems. These include calcium-dependent excitability and mitochondrial vulnerability (Sanchez-Padilla et al., 2014; Surmeier et al., 2017), as well as the energetic demands associated with maintaining extensive axonal arborizations, the latter having been modeled particularly in substantia nigra dopaminergic neurons (Pissadaki and Bolam, 2013). Building on this literature, the present review focuses on how these cellular and axonal constraints may interact with chronic stress-related LC activation and thereby contribute to persistent impairment of dynamic LC-NE regulation.

Chronic stress, defined here as prolonged or insufficiently resolved stress exposure, represents a physiologically relevant condition under which sustained engagement of the LC-NE system is expected to occur. Importantly, such functional alterations may emerge before overt neuronal loss becomes apparent, suggesting that physiological dysregulation can precede structural degeneration. An additional and often underappreciated aspect of LC-NE dysfunction is its potential persistence once established. The combination of sustained physiological load, impaired cellular homeostasis, and vulnerability of long-range axonal projections may contribute to a prolonged state of functional vulnerability (Evans et al., 2022). Such mechanisms could help explain the prolonged course, incomplete recovery, and vulnerability to relapse that characterize many stress-related conditions.

Specifically, this article examines how sustained activation of LC neurons may initiate a multi-level cascade involving cellular strain, mitochondrial stress, axonal vulnerability, and disruption of noradrenergic signaling. The central premise of this review is that chronic stress-related LC-NE dysfunction may be better understood as a loss of dynamic neuromodulatory regulatory capacity—an impaired ability to flexibly adjust tonic and phasic signaling and noradrenergic output according to behavioral context—than as a simple quantitative increase or decrease in norepinephrine signaling (Aston-Jones and Cohen, 2005). By integrating systems-level stress physiology with cellular and axonal mechanisms, the review examines how chronic stress may progressively constrain this regulatory flexibility and contribute to a persistent state of LC-NE rigidity.

## 2 Stress-induced LC activation: CRF-driven tonic load

The proposed pathophysiological cascade begins with prolonged or insufficiently resolved stress exposure, which engages neurobiological stress systems that converge on the locus coeruleus (LC), resulting in sustained alterations in LC activity (McEwen, 2007; Ross and Van Bockstaele, 2021; Valentino and Van Bockstaele, 2008). Central to this process is corticotropin-releasing factor (CRF), which provides a major excitatory drive to LC neurons under conditions of stress (Curtis et al., 2002; Valentino et al., 1992). CRF-containing afferents reach LC dendrites from several stress-responsive regions, including the central nucleus of the amygdala, bed nucleus of the stria terminalis, Barrington's nucleus, and paraventricular nucleus of the hypothalamus (Borodovitsyna et al., 2018; Valentino and Van Bockstaele, 2008), providing an anatomical substrate through which emotional and physiological stress can influence LC excitability and activity states.

Recent circuit-level evidence further supports the concept that LC activity is dynamically regulated by multiple excitatory stress-responsive afferent systems, among which the ventrolateral periaqueductal gray provides a well-characterized example. Wang et al. (2026) identified a glutamatergic projection from the ventrolateral periaqueductal gray to LC noradrenergic neurons that bidirectionally regulated arousal state and pupil size in a frequency-dependent manner. Low-frequency activation preferentially induced anxiety-like behavioral states, whereas higher-frequency stimulation shifted behavior toward panic-like hyperarousal. The authors further demonstrated that inhibition of this pathway reduced anxiety-related avoidance behaviors and lowered baseline arousal. Together, these findings demonstrate that excitatory afferent pathways can dynamically regulate LC-dependent arousal and support the broader view that stress-related LC activation emerges from interactions among multiple afferent systems rather than from CRF signaling alone.

Acute activation of the LC-NE system is adaptive and promotes heightened vigilance, arousal, and behavioral readiness (Aston-Jones and Cohen, 2005; Sara, 2009). Under stress, CRF-mediated excitation can shift LC activity toward increased tonic firing and reduced phasic sensory responsiveness, although the magnitude and persistence of this shift are likely to depend on stress paradigm, duration, and context (Borodovitsyna et al., 2018; Curtis et al., 2012; Slavova et al., 2024). Rather than representing a simple increase in neuronal activity, this transition reflects a shift in the operational mode of the LC-NE system toward a more persistent neuromodulatory state (Grimm et al., 2024; Poe et al., 2020). In this framework, persistent stress-related tonic activation constitutes the initiating physiological load that drives the subsequent cellular and axonal processes described below.

At the intracellular level, CRFR1 activation engages signaling pathways that can link acute excitation to longer-term cellular adaptation. CRFR1 coupling to Gs increases cAMP/PKA signaling, reducing potassium conductance and promoting LC neuronal depolarization, while additional signaling through MAPK/ERK pathways can influence transcriptional and structural responses (Borodovitsyna et al., 2018). In organotypic LC cultures, CRF-induced neurite outgrowth required PKA, MAPK, and Rac1 signaling, providing direct evidence that CRF can engage pathways controlling cytoskeletal remodeling (Swinny and Valentino, 2006). Consistent with longer-term transcriptional effects in vivo, repeated stress increases ERK and CREB phosphorylation in LC neurons, including co-localization of pERK and pCREB after repeated restraint stress (Hebert et al., 2005; Kwon et al., 2006). Together, these findings provide a mechanistic link between CRF-sensitive intracellular signaling, stress-induced transcriptional regulation, and structural plasticity within the LC.

At the cellular level, persistent LC activation imposes a continuous load on LC neurons. Persistent firing increases intracellular calcium influx, elevates metabolic demand, and imposes continuous requirements for maintenance of ion gradients and neurotransmitter cycling (Rangaraju et al., 2014; Sanchez-Padilla et al., 2014; Surmeier et al., 2017). While these processes are integral to normal neuronal function, their prolonged engagement under conditions of chronic stress and insufficient recovery creates a state in which baseline neuronal activity itself becomes a source of cumulative physiological strain.

Importantly, CRF-mediated activation interacts with intrinsic properties of LC neurons that favor stabilization of elevated activity states. LC neurons are intrinsically active and operate close to a threshold of continuous firing, such that sustained excitatory drive can shift the system toward a persistently elevated baseline state (Janitzky, 2020; Valentino and Van Bockstaele, 2008). Over time, sustained excitatory input is likely to stabilize elevated tonic activity and reduce the adaptability of LC state transitions.

Autoregulatory mechanisms further shape this response. Increased norepinephrine release during stress engages inhibitory α2-autoreceptor-mediated feedback pathways that hyperpolarize LC neurons and reduce neuronal excitability (Aghajanian and VanderMaelen, 1982; Valentino and Van Bockstaele, 2008). Although such mechanisms initially serve protective and homeostatic functions, prolonged engagement could paradoxically contribute to reduced responsiveness and diminished phasic signaling capacity (Janitzky, 2020; Ross and Van Bockstaele, 2021). In this context, regulatory mechanisms that are initially adaptive can gradually become incorporated into a stabilized but functionally suboptimal operating state. Additional molecular adaptations may contribute to persistence of altered LC function after repeated stress. Endogenous opioid signaling provides an opposing inhibitory influence on stress-activated LC neurons and has been implicated in restoration of tonic LC activity toward baseline after stressor termination, suggesting that disruption of this counter-regulatory mechanism could also favor persistence of elevated LC activity (Curtis et al., 2012). Stress and sustained CRF signaling can modify CRFR1 trafficking and thereby alter subsequent receptor responsiveness, while repeated stress engages transcriptional pathways capable of changing the molecular phenotype of LC neurons (Borodovitsyna et al., 2018; Hebert et al., 2005). Repeated immobilization stress has also been associated with reduced glucocorticoid receptor mRNA and increased tyrosine hydroxylase mRNA in the LC, suggesting altered glucocorticoid regulation together with enhanced capacity for norepinephrine synthesis (Makino et al., 2002). These mechanisms provide several potential routes through which repeated stress could produce LC adaptations that outlast the initiating stress response.

Support for stress-induced stabilization of elevated LC activity also comes from recent experimental studies. Using a chronic restraint stress model, Jiang et al. (2025) demonstrated persistent activation of noradrenergic neurons in the locus coeruleus accompanied by increased norepinephrine signaling in the medial prefrontal cortex. Pharmacological suppression of LC activity through α2-adrenergic receptor activation reduced neuronal hyperactivity within the LC and normalized stress-associated norepinephrine signaling in the mPFC, supporting an important role for autoregulatory α2-mediated mechanisms in limiting stress-induced LC overactivation.

Importantly, chronically elevated LC activity does not remain limited to altered firing dynamics but initiates a broader cascade of downstream effects that challenge cellular homeostasis within LC neurons. The continuous metabolic and ionic demands associated with elevated activity interact closely with mitochondrial function, oxidative balance, intracellular calcium regulation, and energetic reserve capacity (Rangaraju et al., 2014; Surmeier et al., 2017). While these processes initially support adaptive increases in neuronal activity during stress, their prolonged engagement is likely to gradually compromise the ability of LC neurons to maintain stable physiological regulation over time.

Within the proposed framework, persistent or recurrent stress-related LC activation is considered an initiating physiological load that may engage multi-level cellular and axonal processes across the LC-noradrenergic system. Repeated or sustained increases in LC activity could impose cumulative cellular and energetic demands that progressively constrain regulatory flexibility and thereby contribute to LC-NE rigidity. The cellular and axonal mechanisms contributing to this proposed LC-NE dysfunction model are summarized in Figure 1 and described in the following sections.

**Figure 1:**
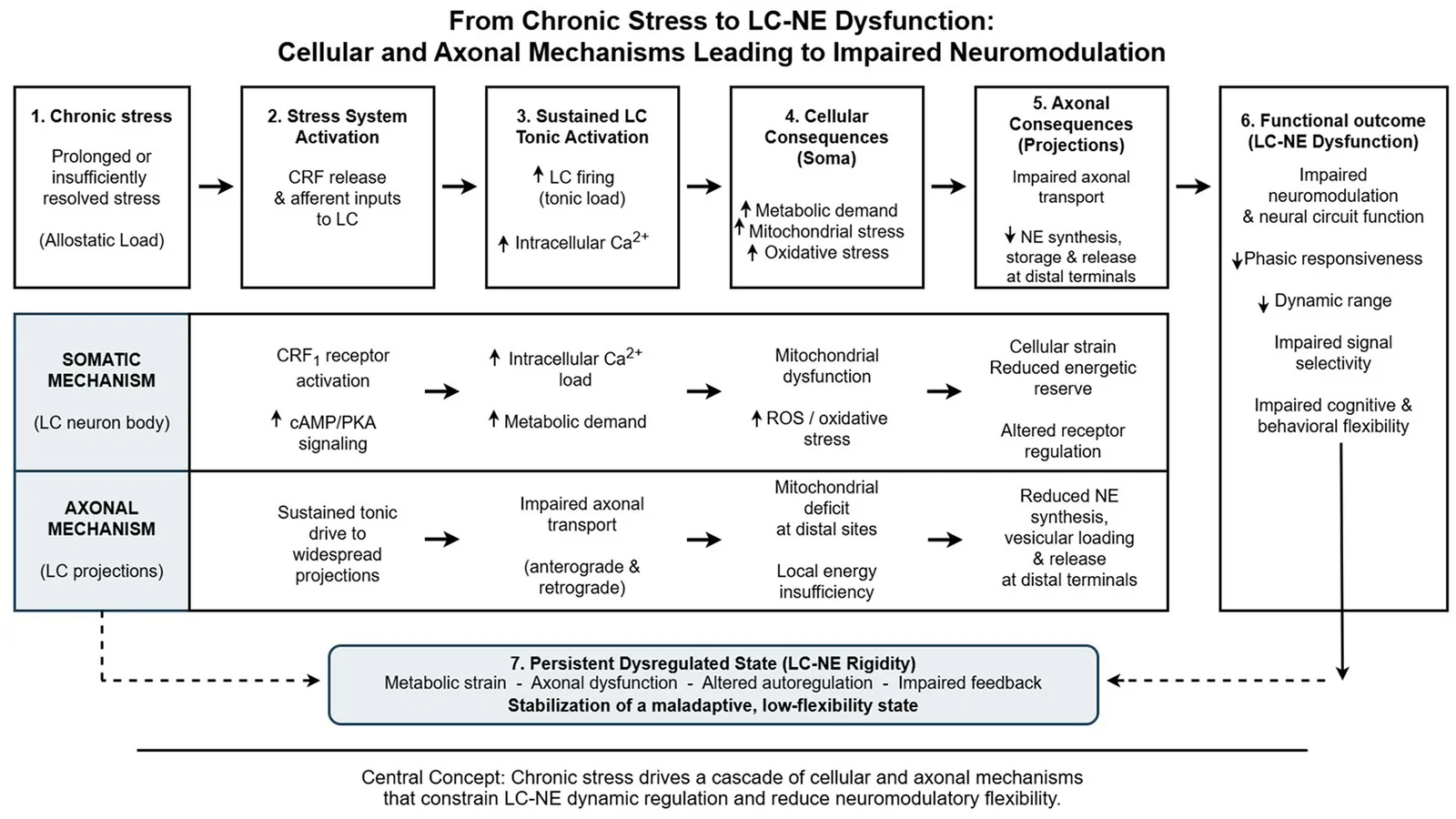
Proposed pathophysiological cascade linking chronic stress to LC-NE dysfunction. Prolonged stress exposure and sustained corticotropin-releasing factor (CRF)-mediated activation impose a persistent tonic load on locus coeruleus (LC) neurons. Due to the intrinsic properties of LC neurons, including autonomous pacemaking activity, high metabolic demand, and extensive axonal arborization, chronic tonic activation is proposed to promote cumulative cellular and axonal strain involving mitochondrial dysfunction, oxidative stress, impaired intracellular transport, and reduced efficiency of distal norepinephrine signaling. These interacting processes are proposed to impair tonic-phasic regulation, progressively reducing phasic responsiveness and constraining the range of LC-NE neuromodulation. At the systems level, such changes could contribute to reduced network flexibility, impaired attentional regulation, cognitive dysfunction, and persistent maladaptive hyperarousal. The model further proposes that LC-NE dysfunction can persist beyond the period of active stress exposure due to stabilization of a low-flexibility neuromodulatory state characterized by reduced adaptability, impaired dynamic regulation, and diminished capacity for context-dependent state transitions. Arrows denote proposed rather than directly demonstrated relationships; the strength of evidence underlying each link varies and is detailed in the corresponding sections of the main text.

## 3 Cellular vulnerability of LC neurons under chronic stress

Following repeated or sustained stress-related recruitment of LC circuits (Figure 1), LC neurons may become exposed to increased cellular and metabolic demands (Evans et al., 2022; Janitzky, 2020; Sanchez-Padilla et al., 2014; Surmeier et al., 2017). While increased activity initially serves adaptive functions, prolonged activation interacts with intrinsic properties of LC neurons to create conditions of cumulative cellular strain. These neurons are characterized by autonomous pacemaking activity, high metabolic demand, and extensive neuromodulatory projections, features that together are likely to reduce their capacity to tolerate sustained physiological stress (Evans et al., 2022; Matschke et al., 2015; Sanchez-Padilla et al., 2014). These features are considered here as interacting rather than independent sources of cellular vulnerability.

A defining feature of LC neurons is their autonomous pacemaking activity, which is maintained through persistent ionic flux and, in particular, calcium-dependent excitability (Sanchez-Padilla et al., 2014; Surmeier et al., 2017). This continuous activity supports stable neuromodulatory tone and rapid responsiveness to salient stimuli. However, it also results in sustained intracellular calcium entry, placing a constant load on intracellular buffering systems and mitochondria. Importantly, mitochondrial oxidant stress in LC neurons appears to depend not only on firing frequency, but also on the specific ionic mechanisms through which elevated activity is generated, particularly the engagement of L-type calcium channels (Sanchez-Padilla et al., 2014). Under conditions in which stress produces sustained or recurrent increases in LC activity, cumulative calcium-handling demands could increase, placing additional strain on intracellular homeostatic mechanisms (Sanchez-Padilla et al., 2014; Surmeier et al., 2017).

Mitochondria play a central role in maintaining cellular stability under these conditions by coupling calcium influx to oxidative phosphorylation and ATP production. This coupling enables LC neurons to sustain high levels of activity but also leads to continuous production of reactive oxygen species during mitochondrial activity. Experimental studies further suggest that calcium entry through L-type channels could contribute directly to mitochondrial oxidant stress through activity-dependent nitric oxide signaling and mitochondrial calcium loading (Sanchez-Padilla et al., 2014). Over time, this is likely to result in an imbalance between energy production and cellular stress defense mechanisms (Evans et al., 2022; Surmeier et al., 2017). One candidate mechanism regulating mitochondrial antioxidant capacity is SIRT3, a mitochondrial deacetylase that modulates manganese superoxide dismutase (MnSOD/SOD2) activity. In substantia nigra dopaminergic neurons, Sirt3 deletion increased MnSOD acetylation, reduced MnSOD activity, and increased mitochondrial oxidative stress (Shi et al., 2017). LC-specific evidence comes from a long-term intermittent hypoxia model, in which reduced SIRT3 and SOD2 expression in LC neurons was associated with increased superoxide production (Zhang et al., 2020). Together with evidence that activity-dependent L-type Ca^2+^ channel opening drives mitochondrial oxidant stress in LC neurons (Sanchez-Padilla et al., 2014), these findings identify SIRT3-dependent antioxidant regulation as a plausible modifier of LC mitochondrial vulnerability. Whether chronic stress itself impairs this mechanism in LC neurons remains unknown. When antioxidant systems are unable to fully compensate, oxidative stress can accumulate, affecting proteins, lipids, and nucleic acids and thereby compromising cellular function (Evans et al., 2022; Surmeier et al., 2017).

In parallel, sustained activation places demands on cellular systems responsible for maintaining protein integrity and turnover. The degradation of damaged or misfolded proteins depends on cellular clearance mechanisms that are sensitive to energetic and oxidative conditions. Under chronic metabolic strain, the efficiency of these processes is likely to decline, leading to the gradual accumulation of dysfunctional proteins and a reduced capacity to maintain normal protein homeostasis (Evans et al., 2022; Surmeier et al., 2017).

The energetic requirements of LC neurons extend beyond baseline pacemaking activity. Maintenance of ion gradients, neurotransmitter synthesis, vesicular loading, and synaptic cycling all contribute to a high and continuously active metabolic profile. During prolonged stress, the increase in tonic firing further elevates ATP demand, reducing the margin between energy supply and demand (Evans et al., 2022; Pissadaki and Bolam, 2013; Sanchez-Padilla et al., 2014; Surmeier et al., 2017). In such a state, even minor impairments in mitochondrial function or substrate availability can lead to a relative energy deficit, with downstream consequences for neuronal excitability and signaling capacity (Evans et al., 2022; Pissadaki and Bolam, 2013).

An important aspect of this vulnerability is that it does not necessarily result in immediate neuronal loss. Instead, LC neurons may enter a state of functional compromise in which they remain structurally intact but operate under physiologically compromised conditions. This state is likely to be characterized by reduced responsiveness to synaptic input, altered firing patterns, and diminished capacity for activity-dependent modulation (Janitzky, 2020). In this context, cellular vulnerability should be understood as a reduction in functional reserve rather than an abrupt transition to degeneration (Poe et al., 2020; Toyoda et al., 2025).

Sustained cellular stress can also affect the mechanisms that normally help stabilize LC activity. Recent experimental findings suggest that chronic stress can weaken the neurons' own inhibitory feedback systems. Toyoda et al. (2025) showed that repeated stress exposure reduced the effectiveness of α2A-adrenergic autoreceptors, leading to persistent overactivation of LC neurons and increased intracellular noradrenergic stress. These findings provide a biologically plausible mechanism through which prolonged activation could gradually destabilize tonic-phasic regulation and reinforce maladaptive patterns of LC activity. Over time, weakened autoregulatory restraint could favor less flexible LC activity states and increase susceptibility to persistent dysregulation (Janitzky, 2020; Valentino and Van Bockstaele, 2008). In this way, mechanisms that are initially adaptive during acute stress exposure may drive the LC-NE system toward a more rigid and less dynamically responsive mode of regulation.

Taken together, the processes described above are unlikely to operate independently. Persistent LC activity increases cumulative calcium-handling and energetic demands, while activity-dependent engagement of L-type Ca^2+^ channels can promote mitochondrial oxidant stress (Sanchez-Padilla et al., 2014). If antioxidant capacity becomes insufficient—including, potentially, SIRT3-dependent defenses—oxidative and metabolic strain may further compromise ATP-dependent calcium buffering, ion-gradient maintenance, protein homeostasis, and other cellular recovery processes. In parallel, chronic stress-induced internalization and downregulation of α2A-adrenergic autoregulatory signaling can weaken an important inhibitory constraint on LC excitability (Toyoda et al., 2025). These processes therefore provide the basis for a potential feed-forward relationship in which sustained activity progressively reduces the cellular reserve required to tolerate continued activity. This proposed interaction represents an integrative hypothesis; the complete causal sequence has not been demonstrated within LC neurons under chronic stress.

These mechanisms may also have relevance beyond stress-related dysfunction. Activity-dependent mitochondrial oxidant stress and impaired antioxidant defenses have been implicated in the selective vulnerability of catecholaminergic neurons in age-related neurodegenerative disorders, including Parkinson's and Alzheimer's disease (Evans et al., 2022; Sanchez-Padilla et al., 2014; Shi et al., 2017). These parallels do not imply a shared or inevitable progression from chronic stress to neurodegeneration, but they identify mitochondrial and metabolic resilience as convergent domains of cellular vulnerability.

Importantly, the cellular processes described above are unlikely to remain confined to the soma. Because LC neurons maintain one of the most extensive projection systems in the central nervous system, persistent disruption of cellular homeostasis is expected to propagate throughout the axonal arbor of the LC-noradrenergic system. The maintenance of axonal transport, mitochondrial distribution, and coordinated neurotransmitter release depends critically on somatic metabolic integrity. Consequently, disturbances in cellular energy balance are likely to preferentially affect distal axonal regions, where energetic reserve capacity is limited and transport distances are greatest. Computational and theoretical models from other highly arborized neuromodulatory systems suggest that such organizational features could predispose to progressive and spatially distributed disruption of neurotransmitter signaling across widespread brain regions (Evans et al., 2022; Pissadaki and Bolam, 2013).

## 4 Axonal vulnerability, volume transmission, and noradrenergic supply failure

Whereas the previous section focused on cellular vulnerability at the level of the soma, the present section examines how these constraints propagate throughout the axonal architecture of the LC-NE system. Axons represent a particularly important site of vulnerability because effective noradrenergic neuromodulation depends not only on neuronal firing, but also on the integrity of a highly extensive and metabolically demanding projection network.

A defining anatomical feature of LC neurons is their extensive projection architecture, with noradrenergic fibers extending throughout large portions of the forebrain, including cortex, hippocampus, thalamus, cerebellum, and spinal cord (Chandler et al., 2014; Schwarz et al., 2015; Swanson and Hartman, 1975). This architecture enables the LC-NE system to exert widespread neuromodulatory influence across neural systems rather than mediating localized point-to-point signaling (Aston-Jones and Cohen, 2005; Grimm et al., 2024; Poe et al., 2020). LC projections are characterized by extensive branching, numerous axonal varicosities, and substantial reliance on volume transmission, in which norepinephrine diffuses through the extracellular space to influence both synaptic and extrasynaptic targets (Agnati et al., 2010; Descarries et al., 1977). Because effective neuromodulation depends on coordinated neurotransmitter release across axonal fields, even modest impairments in axonal transport, mitochondrial function, or neurotransmitter release can substantially reduce noradrenergic signaling efficiency across widespread brain regions.

Axons are particularly sensitive to disturbances in energy supply and intracellular transport. Maintenance of LC projections requires continuous delivery of mitochondria, synaptic vesicles, enzymes, and structural proteins over long distances. These processes depend on efficient intracellular transport mechanisms and are highly energy-dependent (Maday et al., 2014; Sheng, 2017). Under conditions of chronic stress-associated metabolic stress, axonal transport could become compromised, particularly in distal regions that depend heavily on continuous mitochondrial delivery and long-range intracellular transport (Evans et al., 2022; Sheng, 2017).

Extensive arborization increases total axonal length and surface area, while the high density of varicosities requires continuous vesicular cycling and neurotransmitter turnover. In addition, unmyelinated segments necessitate continuous ion flux and Na^+^/K^+^-ATPase activity, resulting in reduced energetic efficiency compared to myelinated fibers. In a computational model of substantia nigra dopaminergic neurons, the energetic cost of action potential propagation increased disproportionately with the size and complexity of the axonal arbor; whether the same quantitative relationship applies to LC neurons has not been directly established (Pissadaki and Bolam, 2013).

At the level of neurotransmission, mitochondrial integrity within axonal terminals is critical for sustaining norepinephrine release. Neurotransmitter release requires tightly regulated calcium dynamics and sufficient ATP supply for vesicle handling and neurotransmitter release processes. Impairment of mitochondrial function in distal regions is likely to directly reduce the efficiency and reliability of norepinephrine release, particularly under conditions requiring phasic activation (Evans et al., 2022; Rangaraju et al., 2014; Sheng, 2017). In addition to energy-related mechanisms, vesicular norepinephrine stores exist in a dynamic equilibrium in which passive leakage into the cytoplasm is counterbalanced by active sequestration through vesicular monoamine transporters. Cytoplasmic norepinephrine that escapes vesicular sequestration is available for MAO-mediated metabolism, generating the reactive aldehyde DOPEGAL (Eisenhofer et al., 2004). In experimental models of Alzheimer's disease, DOPEGAL has been shown to promote oxidative stress, AEP activation, pathological Tau cleavage and aggregation, and LC neurotoxicity (Kang et al., 2020). Whether this pathway contributes to LC dysfunction during chronic stress remains unknown. Collectively, these factors place LC axons under conditions of high metabolic demand and limited energetic reserve. As a result, even moderate disturbances in mitochondrial function, oxidative balance, or transport efficiency can lead to dysfunction (Evans et al., 2022). The exceptional anatomical extent of LC projections is likely to confer particular vulnerability to disturbances in axonal transport and energy homeostasis. Experimental and neurodegenerative models indicate that distal axonal dysfunction and so-called “dying-back” degeneration often precede neuronal cell death, with synaptic and axonal impairment developing progressively before overt somatic degeneration becomes apparent (Adalbert and Coleman, 2013; Coleman, 2005). Evidence from Alzheimer's and Parkinson's disease provides proof-of-principle that noradrenergic axons, terminals, and norepinephrine levels in LC projection regions can decline before substantial loss of LC cell bodies (Weinshenker, 2018). Whether a comparable functional-before-structural dissociation occurs during chronic stress has not been directly established. In the present framework, early impairment of noradrenergic output is therefore proposed as a possible consequence of axonal dysfunction rather than as an established feature of chronic stress-related LC dysregulation.

Within the proposed framework, axonal dysfunction provides one possible mechanism by which LC firing activity could become dissociated from effective noradrenergic output. If axonal transport, terminal integrity, or neurotransmitter release were impaired, tonic LC activity could remain elevated while neuromodulatory influence at projection sites declined. Such a dissociation has not been demonstrated directly during chronic stress and is therefore presented here as a testable mechanistic hypothesis. Within this model, elevated LC activity would therefore not necessarily translate into effective neuromodulatory influence at target regions. Under such conditions, increased norepinephrine turnover could simultaneously reflect elevated neuronal activity and increased intracellular metabolic burden. Excessive catecholamine metabolism is likely to contribute directly to progressive impairment of neuromodulatory signaling through intracellular oxidative and aldehyde-related stress (Eisenhofer et al., 2004). Under conditions of prolonged stress exposure, this mismatch could contribute to reduced phasic responsiveness and impaired signal-to-noise modulation, consistent with principles of tonic-phasic LC regulation described by Aston-Jones and Cohen (2005) and further developed in more recent models of LC dysregulation (Grimm et al., 2024). Thus, the proposed framework allows for a state in which LC electrophysiological activity and effective distributed noradrenergic output become partially dissociated, without assuming that this pattern is a uniform or experimentally established consequence of chronic stress.

The persistence of axonal dysfunction is of particular importance because restoration of effective noradrenergic signaling depends not only on normalization of somatic activity, but also on re-establishment of efficient axonal transport, mitochondrial distribution, and coordinated neurotransmitter release across extensive projection fields. Given the scale and complexity of LC arborization, such recovery processes are likely to occur slowly and may remain incomplete (Evans et al., 2022; McEwen, 2007; Ross and Van Bockstaele, 2021). Importantly, experimental studies suggest that early axonal and synaptic alterations are not necessarily irreversible. Axonal swelling, transport disturbances, and synaptic dysfunction can precede permanent degeneration and may remain partially reversible if cellular homeostasis can be sufficiently restored before structural degeneration becomes fully established (Coleman, 2005).

One candidate mechanism regulating the transition from reversible axonal dysfunction to irreversible degeneration is the NMNAT2–SARM1 pathway. NMNAT2 is a labile axonal survival factor required for local NAD^+^ maintenance; disruption of axonal transport can reduce axonal NMNAT2 levels, increasing the NMN:NAD^+^ ratio and activating the NADase SARM1, which promotes metabolic collapse and axonal degeneration (Hinz and Hoogenraad, 2026). Chronic stress-associated impairment of axonal transport could therefore plausibly increase vulnerability to SARM1-dependent degeneration. However, this pathway has not been directly examined in stress-exposed LC neurons and is proposed here as a candidate mechanism rather than an established component of chronic stress-induced LC pathology.

These observations identify the axonal domain as a critical locus of vulnerability within the LC-NE system. The combination of diffuse volume transmission, high metabolic demand, and dependence on long-range intracellular transport renders LC projections particularly sensitive to chronic stress-induced cellular strain. Importantly, these mechanisms could create a dissociation between neuronal activity and effective neuromodulatory output. Within the proposed framework, LC neurons could remain tonically active while the efficiency, spatial precision, and temporal coordination of norepinephrine signaling deteriorate across distributed projection fields.

In this state, effective noradrenergic function depends not only on neuronal firing itself, but on the integrity of a large-scale release system spanning extensive anatomical and functional domains (Agnati et al., 2010; Evans et al., 2022; Maday et al., 2014). Consequently, disturbances in axonal integrity and neurotransmitter release could progressively translate into impaired regulation of distributed brain networks at the systems level.

## 5 Noradrenergic dysfunction and loss of dynamic modulation

The cellular and structural mechanisms described in the preceding sections converge on a common functional outcome: impaired noradrenergic signaling and reduced capacity for neuromodulation within the LC-NE system. Although chronic stress initially increases activation of locus coeruleus (LC) neurons, sustained tonic load, cellular strain, and axonal dysfunction progressively alter the functional properties of the LC-noradrenergic system (Evans et al., 2022; Surmeier et al., 2017; Valentino and Van Bockstaele, 2008). Importantly, this transition cannot be adequately understood in terms of overall firing rate alone, but rather by reduced capacity for temporally precise, context-dependent, and phasic modulation of neural activity (Aston-Jones and Cohen, 2005; Grimm et al., 2024). From this perspective, seemingly divergent manifestations of LC-NE dysfunction—including excessive activation, reduced responsiveness, altered autoregulatory or postsynaptic receptor signaling, and structural or axonal impairment—can be understood as different expressions of a shared loss of adaptive neuromodulatory regulation rather than as mutually exclusive states.

A central feature of this transition is altered interaction between tonic and phasic LC activity. Under physiological conditions, tonic LC activity contributes to vigilance regulation, while transient phasic responses occur in relation to salient or behaviorally relevant events (Bouret and Sara, 2005; Rajkowski et al., 1994). This organization enables selective enhancement of signal processing in response to behaviorally relevant stimuli while maintaining overall network stability (Aston-Jones and Cohen, 2005; Grimm et al., 2024). Under acute predator stress, increased tonic LC discharge is accompanied by reduced auditory-evoked phasic discharge and a lower signal-to-noise ratio, with CRF signaling playing a major causal role in this shift (Curtis et al., 2012). Whether an analogous tonic-phasic alteration persists during chronic stress has not been directly established. Persistent high-tonic states are therefore considered here as a potential constraint on phasic responsiveness rather than as a uniformly demonstrated consequence of chronic stress (Aston-Jones and Cohen, 2005; Janitzky, 2020).

As discussed in previous sections, this reduction in phasic signaling is likely to arise not only from altered electrophysiological properties at the level of the soma, but also from impaired neurotransmitter release, disrupted axonal transport, and reduced spatial coordination of norepinephrine signaling across projection systems.

These changes alter both the timing and spatial organization of noradrenergic signaling. Sustained tonic activity is likely to promote more continuous and spatially diffuse norepinephrine signaling across widespread projections, consistent with principles of volume transmission (Agnati et al., 2010), while impaired phasic responses reduce stimulus-linked modulation (Aston-Jones and Cohen, 2005). The result is a less selective signaling state in which the distinction between baseline activity and transient stimulus-driven responses becomes reduced. Impaired phasic release further limits the system's ability to generate temporally focused increases in norepinephrine at target sites, reducing the precision of neuromodulatory control over neural circuits (Grimm et al., 2024).

This loss of precision is particularly relevant in prefrontal cortical networks, where noradrenergic signaling contributes to working memory, attentional regulation, and executive control (Arnsten, 2009; Ramos and Arnsten, 2007). Under conditions of prolonged tonic drive and impaired phasic signaling, these networks could become more susceptible to interference and less capable of maintaining stable delay-related and task-relevant activity (Birnbaum et al., 2004; Jiang et al., 2025; Wang et al., 2007). Such conditions are likely to reduce the stability and selectivity of prefrontal cortical network activity by disrupting the recurrent excitatory connectivity required for sustained working memory and attentional regulation (Wang et al., 2026). From this perspective, cognitive impairments associated with chronic stress may reflect impaired neuromodulatory regulation rather than simple reductions in norepinephrine availability.

At the receptor level, these effects can be amplified by altered adrenergic receptor engagement. At moderate norepinephrine concentrations, high-affinity α2-adrenergic receptors contribute to stabilization of prefrontal network activity (Arnsten, 2009; Wang et al., 2007). However, under conditions associated with sustained stress-related norepinephrine release, lower-affinity α1- and β-adrenergic receptors become increasingly engaged (Ramos and Arnsten, 2007). Activation of these receptors is associated with reduced network stability, thereby further impairing signal discrimination and cognitive performance (Birnbaum et al., 2004; Ramos and Arnsten, 2007). At higher catecholamine concentrations, excessive cAMP signaling can weaken recurrent prefrontal network connectivity through activation of membrane ion channels that reduce persistent neuronal firing, thereby impairing the activity patterns required for executive regulation and working memory (Wang et al., 2007).

Autoregulatory mechanisms could further contribute to this functional state. Sustained increases in norepinephrine release engage inhibitory feedback pathways, including α2-adrenergic autoreceptors on LC neurons (Aghajanian and VanderMaelen, 1982; Valentino and Van Bockstaele, 2008). Although these mechanisms normally limit excessive activation, prolonged engagement is likely to contribute to reduced responsiveness and diminished capacity for phasic discharge (Ross and Van Bockstaele, 2021).

These alterations reflect a broader impairment in the temporal and spatial coordination of neuromodulatory signaling. Noradrenergic projections normally influence activity across neural systems by modulating neuronal responsiveness and facilitating transitions between functional states (Sara, 2009). When phasic neuromodulation becomes less temporally precise under conditions of sustained stress-related tonic activation, network activity can become less adaptable and more dominated by persistent background activation (Grimm et al., 2024). Recent multimodal imaging studies provide additional systems-level support for the concept that optimal LC-NE function depends on preservation of a balanced arousal state. Using combined EEG-fMRI recordings in humans and mice together with causal manipulation of LC-noradrenergic neurons, Tong et al. (2025) demonstrated an inverted U-shaped relationship between arousal state and large-scale network integration. Intermediate arousal levels were associated with maximal functional connectivity, whereas both low and excessively elevated arousal states impaired network integration. These findings support the view that sustained stress-related increases in tonic noradrenergic activity could reduce the efficiency of large-scale neural coordination by disrupting optimal neuromodulatory balance.

Functionally, this may correspond to a shift from flexible, goal-directed regulation toward increased distractibility, impaired attentional regulation, and reduced selectivity of information processing (Arnsten, 2009; Aston-Jones and Cohen, 2005). While such a state can be adaptive during acute threat exposure, sustained activation over longer periods is likely to become maladaptive (Janitzky, 2020; McEwen, 2007).

The consequences of impaired LC-NE modulation extend beyond cognition. Because noradrenergic signaling contributes to autonomic regulation, emotional processing, and energy management, stress-related dysregulation of LC-NE signaling can affect multiple physiological and behavioral domains simultaneously (McEwen, 2007). Reduced capacity for selective neuromodulatory regulation can increase background activity and reduce selectivity in sensory processing, potentially contributing to subjective experiences of overload, fatigue, and impaired attentional filtering. Similarly, impaired coordination between LC output and autonomic control systems could reduce physiological flexibility and adaptive responsiveness.

A key feature of this functional state is its persistence beyond the period of active stress exposure. As described throughout the preceding sections, chronic tonic activation, metabolic strain, altered receptor regulation, and impaired axonal transport can continue to constrain LC-NE function even after external stressors have been reduced or removed. Because restoration of effective neuromodulatory function depends not only on normalization of neuronal firing, but also on recovery of mitochondrial function, intracellular transport, and coordinated neurotransmitter release across extensive projection fields, recovery is likely to occur gradually and may remain incomplete. Consequently, restoration of LC-NE function cannot be understood solely as passive reduction of stress exposure, but is likely to require active re-establishment of dynamic neuromodulatory regulation, including recovery of tonic-phasic interaction and restoration of efficient phasic signaling across projection systems (Ericson, 2026).

Taken together, these observations support a framework in which chronic stress may constrain the flexibility of LC-NE regulation, with the physiological expression of this rigidity potentially varying across stress paradigms, stages, and contexts (Grimm et al., 2024; Janitzky, 2020). Importantly, this transition emerges not from a single pathological mechanism, but from the cumulative interaction of sustained tonic activation, cellular strain, mitochondrial stress, and axonal dysfunction operating across multiple biological levels. Accordingly, stress-related dysfunction may be understood not simply as altered neurotransmitter activity, but as progressive impairment of the system's capacity for dynamic and adaptive neuromodulatory regulation.

## 6 Conclusion

Chronic stress is proposed to alter the functional state of the LC-NE system through prolonged CRF-driven activation and cumulative cellular and axonal strain (Ross and Van Bockstaele, 2021; Valentino and Van Bockstaele, 2008). Because LC neurons combine autonomous pacemaking activity, high metabolic demand, and extensive axonal arborization, prolonged tonic load is proposed to generate progressive vulnerability involving mitochondrial stress, impaired intracellular transport, and reduced efficiency of coordinated norepinephrine signaling (Evans et al., 2022; Pissadaki and Bolam, 2013; Surmeier et al., 2017).

Within this framework, impaired neuromodulation cannot be explained solely by altered firing dynamics. Effective LC-NE function depends on preservation of tonic-phasic dynamics, temporally precise norepinephrine signaling, intact intracellular transport, and adaptive receptor regulation across distributed projection systems. Chronic stress is proposed to progressively constrain these mechanisms, resulting in reduced phasic responsiveness, impaired network flexibility, diminished signal selectivity, and reduced neuromodulatory adaptability. Such changes could contribute to persistent cognitive dysfunction, impaired attentional regulation, and reduced behavioral adaptability.

This perspective reframes stress-related dysfunction as progressive impairment of dynamic neuromodulatory regulation resulting from cumulative stress-related physiological load, rather than as a transient disturbance of stress signaling alone. In this model, prolonged stress exposure is proposed to gradually shift the LC-NE system from a flexible and context-sensitive mode of regulation toward a more constrained functional state characterized by reduced dynamic range and impaired tonic-phasic coordination. Recovery from chronic stress-related LC-NE dysfunction is therefore proposed to depend not only on reduction of external stressors, but also on restoration of the cellular, axonal, and network-level mechanisms that support adaptive LC-NE regulation.
